# The Histrionic Temperament: The Poseur

**Published:** 1918-12-14

**Authors:** 


### HUMAN TEMPERAMENTS.

### THE HISTRIONIC TEMPERAMENT: THE POSEUR.

But all their works they do for to be seen of men.—Matt. xxiii. 5.

Macaulay says, in a well-known passage, that the first William Pitt "had one fault, which of all human faults is most rarely found in company with true greatness. He was extremely affected. He was an almost solitary instance of a man of real genius, and of a brave, lofty, and commanding spirit, without simplicity of character. He was an actor in the Closet, an actor at Council, an actor in Parliament; and even in private society he could not lay aside his theatrical tones and attitudes." Even his most intimate crony could never obtain admission to his chamber "till everything was ready for the representation, till the dresses and properties were all carefully disposed, till the light was turned with Rembrandt-like effect on the head of the illustrious performer, till the flannels had been arranged with the air of a Grecian drapery, and the crutch placed as gracefully as that of Belisarius or Lear." It is a little odd that Macaulay, with his unsurpassed and unsurpassable memory, and writing in 1834, only a few years after the death of Napoleon, "the gleam of whose glory arose and o'ershadowed the earth with its fame," should have spoken of Pitt as an almost solitary instance of habitual histrionic display by a man of real genius. Macaulay was too able a man to have shared the vulgar prejudice of so many English contemporaries of Napoleon, that he was not a man of genius, but a mere histrionic charlatan. Macaulay was too able to make this blunder, and too honest to pretend that he had forgotten the instance. Probably, like the consummate literary artist he was, he deliberately refrained from placing near the object he was depicting another of equal prominence that would have diverted attention from his main theme. A memory such as his could easily have furnished him with other instances also of men of real genius, at any rate of great ability, who were so habitually given to theatrical display as to serve as instances of the Histrionic Temperament.

### Why Napoleon Posed.

And there was a better reason for refusing to cite the instance of Napoleon. Napoleon did frequently pose, no doubt. He did very often adopt the tones and attitudes of the actor, and strut and gesticulate for to be seen of men; but not on that account was he necessarily a Poseur. When Napoleon posed, he posed deliberately and of set purpose in order to achieve an end that he had in view, and that could best be achieved by posing. Most Frenchmen are born actors, in the sense that they can act effectively upon occasion, and every Frenchman appreciates good acting, admires the actor, and esteems him more highly if he acts well. Napoleon was a consummate judge of men, and was fully aware of this trait in the French character, and he took advantage of it. He wished to impress Frenchmen and to be popular amongst them and admired by them, and he neglected no means that were likely to serve his purpose: therefore he posed; and he posed, as he did everything else, effectively and well; but not on that account was he a Poseur.

### Three Variations of Poseurs.

For the Poseur does not pose in order to achieve a purpose. He poses, not deliberately and upon reflection, in order to gain advantage by posing and gesticulating and grimacing. He poses because he cannot help it. It is his nature. He struts and gesticulates and strikes attitudes to impress the spectators, no doubt, but not, as Napoleon did, with any ulterior purpose beyond the mere purpose of producing an impression, attracting attention, and evoking admiration. He poses, not to achieve any outward purpose, but to satisfy an inward craving. It is his nature to come forward and seek to attract attention, just as it is the nature of the shy man to shrink back and elude attention. The two temperaments are antithetically opposed to one another, but each is a true temperament, born with the person who displays it, and not to be overcome without pains and trouble. The true Poseur poses because it is his nature to pose, because to do so is the natural expression of his inward craving, because he can no more help it than that born poseur, the peacock, can help spreading his tail and strutting before his dames, or that other born poseur, the mandril, can help turning away in order to display to the embarrassed spectator its scarlet and cerulean hinder end. Napoleon posed deliberately and of set purpose, in order to achieve an ulterior end. Very likely he enjoyed posing, as we all enjoy doing that that we do well; but he would still have posed even if it had bored him to do so, even if he had been obliged to learn how to do so; and it is said that he did in fact take lessons in deportment from Talma, the leading actor of his time. The true Poseur poses because to him posing is delightful, because he enjoys it, because it exercises a faculty that he possesses in high degree, in overflowing abundance; and the exercise of such a faculty, if we possess one, is a necessity of human nature.

### Self-Consciousness and that Other Consciousness.

Like the shy man, the Poseur possesses in high degree that other-consciousness that is paradoxically called self-consciousness. Both of them are acutely sensitive to the presence and attention of their fellows; but whereas to the shy man the knowledge that he is attracting the attention of others is painful, embarrassing, and paralysing, to the Poseur it is delightful, enjoyable, and stimulating. The one is never at his best unless he is alone, and can do, free from observation and attention, whatever he has to do. The other is never at his best unless he

**Human Temperaments: The Poseur**—(*cont.*)

is in company, and can attract to himself the observation and attention of his fellows. As with certain drugs, that which paralyses one man stimulates the other.

The difference between the unaffected man and the affected man, between the simple straightforward person and the person who is, as Macaulay says, "without simplicity of character," is that the former acts with a single eye to what he is doing: the latter has but one eye on what he is doing, the other being intent upon the spectators, to see how they are impressed by what he is doing. The one acts in order to get the thing done; the other acts partly to get the thing done, but mainly to impress the spectators with his manner of doing it, and with his success in getting it done. All their works they do for to be seen of men.

Unless an action is become automatic from perpetual repetition, the diversion of our attention from what we are doing is apt to deteriorate the work; and even if an action is become thoroughly automatic, though it may be spoilt by paying sedulous attention to its details, it cannot be performed with full efficiency unless attention, great attention, is paid to the general manner of doing. A musician who should try to pay attention to the performance of each individual note would infallibly ruin his performance, but a musician who should allow his attention to be diverted altogether from his performance might execute every individual note faultlessly, but the performance as a whole would be a failure. It would be inartistic. There would be no life in it. The critics would detect the failure at once, and their descriptions of the performance would be disparaging. That work to which we attend most closely is, other things being equal, the best done; and hence the person who rarely gives his whole attention to what he is doing rarely attains to the highest excellence in what he does. His performance is often mediocre, and sometimes altogether fails. In either case, the merit of the performance is out of proportion to the admiration that he asks for by his posing; and for this reason alone he may become an object of contempt.

### Actions which Excite our Ire.

But even if he is not on this ground an object of contempt, the Poseur incurs the disapprobation and disesteem of those who see through his devices and recognise that he is posing. We feel that histrionic display is unworthy. There is something in it that goes against the grain of simple, straightforward people. We feel that there is something derogatory, something despicable, in the art and practice of posing, so that it excites in some degree our contempt, in some degree our ire. Why is this? It is for several good and sufficient reasons. In the first place, the strutting, the gesticulation, the grimacing divert our attention from what we meant to attend to. We wish to attend to what is being done, and between us and the object of our attention something else is interposed, and the interposition is resented. The diversion of attention is resented. We want to attend to the performance, and our attention is constantly being carried away to the antics of the performer. The diversion of attention is annoying, and excites our ire.

In the second place, we regard the posing as a kind of imposture. It is an attempt to obtain admiration by false pretences. We feel that a man's claim to our admiration should stand upon his achievements, and that if his achievements themselves constitute a claim on our admiration, there is no need to claim that admiration, as the Poseur does, on other grounds. In this matter we have a right to form our own judgment, and we resent its being taken out of our hands and prejudged by an interested party. Moreover, we are very apt to suppose that if a man had a legitimate claim to our admiration on the ground of his achievements, then, as there would be no need for a spurious claim, such as posing virtually is, no such spurious claim would be made. The reasoning is not always valid, as the case of the Earl of Chatham proves, but it commends itself to the judgment, and it is very often valid. It is usually valid.

### A Sturdy Beggar.

A man's claim to our admiration should rest upon the solid ground of his achievements, and not upon empty and futile grimaces and posing. If he is content to let it rest upon his achievements, and if his achievements are such as we consider admirable, our admiration and applause are freely given; but we feel that they are free gifts, to be given or withheld at our own discretion and pleasure, and we resent being dunned and importuned for them as the Poseur duns and importunes us by his posing and his antics. We look upon him as a sturdy beggar, who demands as a right that which is our own to bestow or to withhold as we please. In this way also the Poseur incurs our displeasure.

Finally, the natural, simple man shrinks from the limelight glare of public attention. He may not be positively shy, but he has this in common with the shy man—that until he becomes well accustomed to it, he is embarrassed and confused by becoming an object of general attention. He may be flattered, but he is none the less embarrassed, and when he witnesses the embarrassment of another person in these circumstances he is full of sympathy for the victim. But when he discovers a man who does not wait for the limelight to be turned upon him, but seeks it out, attracts it to himself, basks in it, revels in it, poses in it, and struts in it, then he looks upon that man as abnormal, as indeed he is. The normal spectator regards the Poseur with the suspicion, dislike, and contempt with which the stranger and the foreigner are always *primâ facie* regarded. We feel towards him as we feel towards the drunken man and the lunatic, as the townsman feels towards the yokel and the yokel towards the townsman. He is not our sort, and we are much inclined, on that account alone, to heave half a brick at him.

